# Efficacy and safety of levetiracetam as adjunctive therapy for refractory focal epilepsy

**DOI:** 10.1590/0004-282X-ANP-2020-0082

**Published:** 2021-05-08

**Authors:** Maria Luiza Giraldes de MANREZA, Tatiane Amaral PAN, Eduardo Quinalha CARBONE, Antonio Carlos Amedeo VATTIMO, Renata HERRERA, Douglas Costa MORAIS, Rita Antonelli CARDOSO, Glenda Corrêa Borges de LACERDA, Katia LIN, Frederico Nakane NAKANO, Pedro André KOWACS, André Luis Fernandes PALMINI, Adélia Maria de Miranda Henriques SOUZA, Stevin ZUNG, Elza Márcia Targas YACUBIAN

**Affiliations:** 1 Universidade de São Paulo, Faculdade de Medicina, Hospital das Clínicas, Centro de Pesquisa, São Paulo SP, Brazil. Universidade de São Paulo Universidade de São Paulo Faculdade de Medicina Hospital das Clínicas São Paulo SP Brazil; 2 Aché Laboratórios Farmacêuticos S.A., Núcleo de Inovação, Núcleo Médico-Científico, Guarulhos SP, Brazil. Aché Laboratórios Farmacêuticos S.A. Núcleo de Inovação Núcleo Médico-Científico Guarulhos SP Brazil; 3 STATS Estatística em Ciências e Negócios, São Paulo SP, Brazil. STATS Estatística em Ciências e Negócios São Paulo SP Brazil; 4 Universidade Federal Fluminense, Hospital Universitário Antônio Pedro, Rio de Janeiro RJ, Brazil. Universidade Federal Fluminense Universidade Federal Fluminense Hospital Universitário Antônio Pedro Rio de Janeiro RJ Brazil; 5 Universidade Federal de Santa Catarina, Hospital Universitário, Núcleo de Pesquisa em Neurologia Experimental e Clínica, Florianópolis SC, Brazil. Universidade Federal de Santa Catarina Universidade Federal de Santa Catarina Hospital Universitário Núcleo de Pesquisa em Neurologia Experimental e Clínica Florianópolis SC Brazil; 6 Universidade de São Paulo, Faculdade de Medicina de Ribeirão Preto, Hospital das Clínicas, Unidade de Pesquisa Clínica, Ribeirão Preto SP, Brazil. Universidade de São Paulo Universidade de São Paulo Faculdade de Medicina de Ribeirão Preto Hospital das Clínicas Ribeirão Preto SP Brazil; 7 Instituto de Neurologia de Curitiba, Curitiba PR, Brazil. Instituto de Neurologia de Curitiba Curitiba PR Brazil; 8 Pontifícia Universidade Católica do Rio Grande do Sul, Hospital São Lucas, Centro de Pesquisa Clínica, Porto Alegre RS, Brazil. Pontifícia Universidade Católica do Rio Grande do Sul Pontifícia Universidade Católica do Rio Grande do Sul Hospital São Lucas Centro de Pesquisa Clínica Porto Alegre RS Brazil; 9 Instituto de Medicina Integral Prof. Fernando Figueira, Recife PE, Brazil. Instituto de Medicina Integral Professor Fernando Figueira Instituto de Medicina Integral Prof. Fernando Figueira Recife PE Brazil; 10 Universidade Federal de São Paulo, Escola Paulista de Medicina, Departamento de Neurologia e Neurocirurgia, São Paulo SP, Brazil. Universidade Federal de São Paulo Universidade Federal de São Paulo Escola Paulista de Medicina Departamento de Neurologia e Neurocirurgia São Paulo SP Brazil

**Keywords:** Levetiracetam, Refractory Epilepsy, Focal Seizures, Antiseizure Medications, Seizures, Levetiracetam, Epilepsia Refratária, Crises Focais, Fármacos Anticrise, Crises Epilépticas

## Abstract

**Background:**

Epilepsy affects about 50 million people worldwide and around 30% of these patients have refractory epilepsy, with potential consequences regarding quality of life, morbidity and premature mortality.

**Objective:**

The aim of treatment with antiseizure medications (ASMs) is to allow patients to remain without seizures, with good tolerability. Levetiracetam is a broad-spectrum ASM with a unique mechanism of action that differs it from other ASMs. It has been shown to be effective and safe for treating adults and children with epilepsy.

**Methods:**

This was a phase III, multicenter, randomized, double-blind, placebo-controlled trial to evaluate the efficacy and safety of levetiracetam in children and adults (4-65 years) as an adjuvant treatment for focal-onset seizures. It was conducted among 114 patients undergoing treatment with up to three ASMs. The primary efficacy analysis was based on the proportion of patients who achieved a reduction of ≥ 50% in the mean number of focal seizures per week, over a 16-week treatment period. The patients were randomized to receive placebo or levetiracetam, titrated every two weeks from 20 mg/kg/day or 1,000 mg/day up to 60 mg/kg/day or 3,000 mg/day.

**Results:**

Levetiracetam was significantly superior to placebo (p = 0.0031); 38.7% of the participants in the levetiracetam group and 14.3% in the control group shows reductions in focal seizures. Levetiracetam was seen to have a favorable safety profile and an adverse event rate similar to that of placebo.

**Conclusion:**

Corroborating the results in the literature, levetiracetam was shown to be effective and safe for children and adults with refractory focal-onset epilepsy.

## RESUMO

**Introdução:**

A epilepsia afeta cerca de 50 milhões de pessoas em todo o mundo e aproximadamente 30% desses pacientes apresentam epilepsia refratária, com possíveis consequências na qualidade de vida, morbidade e mortalidade prematura.

**Objetivo:**

O objetivo do tratamento com fármacos antiepilépticos (FAEs) é permitir que os pacientes permaneçam sem crises epilépticas com boa tolerabilidade. O levetiracetam (LEV) é um FAE de amplo espectro, com mecanismo de ação único, diferente dos demais e que demonstra ser eficaz e seguro no tratamento de adultos e crianças.

**Métodos:**

Estudo de fase III, multicêntrico, randomizado, duplo-cego e controlado por placebo avalia a eficácia e a segurança do LEV em crianças e adultos (4-65 anos) como tratamento adjuvante para crises de início focal em 114 pacientes já tratados com até três FAEs. A análise de eficácia primária foi baseada na proporção de pacientes que apresentaram redução ≥50% no número médio de crises epilépticas focais semanais, durante 16 semanas. Os pacientes foram randomizados para receber placebo ou LEV, titulado a cada duas semanas de 20 mg/kg/dia ou 1.000 mg/dia até 60 mg/kg/dia ou 3.000 mg/dia.

**Resultados:**

LEV foi significativamente superior ao placebo (p=0,0031), com 38,7% dos participantes no grupo LEV e 14,3% no grupo controle que apresentaram redução das crises focais. LEV apresenta bom perfil de segurança com eventos adversos semelhantes ao placebo.

**Conclusão:**

Corroborando com os resultados da literatura, o levetiracetam mostra-se eficaz e seguro para crianças e adultos com epilepsia focal refratária.

## INTRODUCTION

Epilepsy is one of the most common neurological diseases and affects approximately 50 million people worldwide^1^^,^^2^. It is characterized by recurrent epileptic seizures caused by excessive and synchronous neuronal discharges^1^. Individuals with epilepsy are more susceptible to physical trauma (such as fractures and bruises), psychiatric disorders (such as depression and anxiety) and premature death. The risk that they face is up to three times higher than that of the general population^2^.

The aim of antiseizure medications (ASMs) is to combine seizure prevention with good drug tolerability^3^. However, around 30% of patients have refractory epilepsy^4^, which is particularly common in individuals with focal seizures^5^. This makes refractory epilepsy a high-cost health problem and a major concern for patients, families and society in general^4^.

For treating refractory epilepsy, there is an expectation that new ASMs can be developed^6^ or that effective combinations of two or more existing treatments can be found^1^. Several combinations of ASMs can be used to achieve this purpose, leading to different success rates and tolerability profiles.

Levetiracetam (LEV) is a broad-spectrum ASM with a unique mechanism of action that make it one of the most commonly prescribed drugs of its class. It is recommended as a first-line add-on agent for focal seizures and, additionally, has a favorable pharmacokinetic profile. Studies have shown that LEV is an effective anti-seizure medication for both adults and children with generalized or focal-onset refractory seizures, at doses of 1,000-3,000 mg/day or 60 mg/kg/day, with an acceptable adverse event profile^7^. However, little information about LEV use in the Brazilian population is available.

The present clinical study was designed to evaluate the efficacy and safety of LEV, in Brazilian adults and children, as an adjunctive treatment for refractory focal epilepsy.

## METHODS

### Population

This study included participants between 4 and 65 years old with refractory focal epilepsy, with or without focal to bilateral tonic-clonic seizure, as defined by the International League Against Epilepsy (ILAE)^8^, They needed to have had this condition for at least two years, without any progressive or expansive brain injury previously documented, with a minimum of 12 seizures in the last 12 weeks before screening; and they needed to have been on a stable therapeutic regimen of up to three ASMs for at least one month. Women needed to be using contraception and have a negative pregnancy test result throughout the study period.

The exclusion criteria comprised presentation of any of the following: non-epileptic events; psychogenic non-epileptic seizures; ≥ 3 occurrences of subintrant epileptic seizures in the last 12 weeks prior to the study screening visit; cognitive or progressive epileptic syndromes; history of schizophrenia or suicide attempt; severe intellectual disability of any etiology; or clinically significant diseases of hematopoietic, gastrointestinal, cardiovascular, hepatic, renal, neurological, endocrine, psychiatric, autoimmune, pulmonary or other origin, at the discretion of the investigator.

All participants aged 18 years old or above provided written consent prior to undergoing any study procedure. For participants between 12 and 17 years old, consent was obtained from them as well from their legal guardians. For participants under 12 years old, only the consent of legal guardians was obtained.

### Study design

This was a phase III, multicenter, randomized, double-blind, placebo-controlled clinical trial to evaluate the efficacy and safety of levetiracetam as a therapeutic adjunct for controlling focal epileptic seizures (focal aware seizures (IA), focal seizures with impaired awareness (IB) and focal to bilateral tonic-clonic seizures (IC), as defined in the ILAE classification^8^.

This study was conducted at eight research centers in Brazil: three in the state of São Paulo (Universidade Federal de São Paulo [UNIFESP], Hospital das Clínicas da Faculdade de Medicina da Universidade de São Paulo [HC-FMUSP] and Universidade de São Paulo [USP], Ribeirão Preto campus); one in Rio Grande do Sul (Pontifícia Universidade Católica do Rio Grande do Sul [PUC-RS]); one in Paraná (Instituto de Neurologia de Curitiba [INC]); one in Pernambuco (Instituto de Medicina Integral Professor Fernando Figueira [IMIP]); one in Rio de Janeiro (Hospital Universitário Clementino Fraga Filho [HUFF]); and one in Santa Catarina (Universidade Federal de Santa Catarina [UFSC]). The study protocol was approved by the independent ethics committees of each institution.

**Figure 1.**
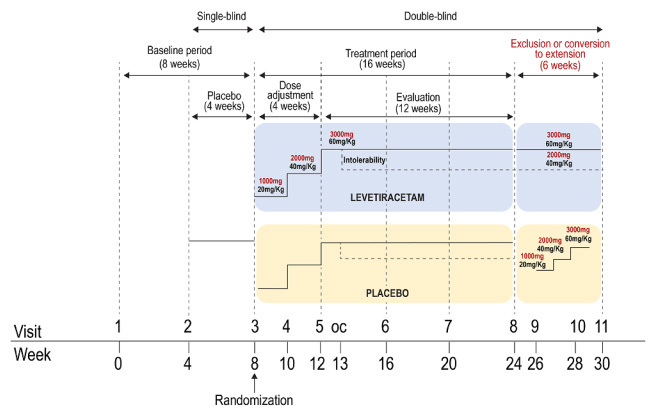
Design of the clinical study.

The total study period comprised 30 weeks, from January 2013 to August 2015, divided into three parts (Figure 1):

OC: optical visit.

Baseline: 8 weeks. The treatment regimen followed by the patient before the study was maintained in the four weeks prior to enrollment. In the last four weeks, it became a simple-blind study with the addition of placebo.

Treatment: 16-week double-blind period. Participants were randomized 1:1 to levetiracetam or placebo, with progressive titration performed every two weeks, starting with 20 mg/kg/day or 1,000 mg/day and going up to 40 mg/kg/day or 2,000 mg/day from the 3^rd^ to 4^th^ week and to 60 mg/kg/day or 3,000 mg/day from the 5^th^ to 20^th^ week. If the participant did not tolerate a higher dose, it could return to 2,000 mg/day or 40 mg/kg/day. If participants did not tolerate this lower dose, they were excluded from the study.

Extension: 6-week double-blind period. For this, participants who agreed to continue taking part in the study were included. Treatment was maintained for the levetiracetam group, and placebo group participants were converted to active treatment, starting with titration every two weeks.

Levetiracetam and placebo products were both available as coated tablets or oral solution, for participants over or below 15 years old, respectively.

Adherence to treatment was verified by counting the tablets or, in the case of oral solution, evaluating the participant's diary that was given to the subject’s parents. This diary was given to all participants in order to register the number of seizures.

Participants who had a serious adverse event, loss of follow-up or adherence, pregnancy, change in baseline therapy for epilepsy or a change in the LEV dose not allowed by the protocol were discontinued from the study.

### Objectives

The primary efficacy variable was a reduction of ≥ 50% in the mean number of focal seizures per week during treatment (4-week dose adjustment + 12-week evaluation) from baseline (4 weeks without study drug + 4 weeks on placebo).

The secondary objectives were to determine the following: the change in the average weekly number of IA, IB and IC seizures; the proportion of the participants with ≥ 50% reduction in the average number of days a week with focal seizures; the proportion of participants with no epileptic seizures during the period; and tolerability of LEV in relation to occurrences of adverse events.

The exploratory endpoint was the quality of life, evaluated through the questionnaires QOLIE-AD-48 (11 to 17 years old) or QOLIE-31 (≥18 years old) and the inventory of depression in neurological disorders for epilepsy (≥ 18 years old)^9^^,^^10^^,^^11^. Student's t test was used for the analyses.

### Safety assessment

The safety assessment for the study was performed based on measurement of adverse event occurrences and on evaluation of clinical examination results, ECG findings and laboratory tests, including blood chemistry (sodium, potassium, TGO, TGP, alkaline phosphatase, total bilirubin and fractions, creatinine, urea, gamma-GT and total protein and fractions), hematology (platelet count and leukogram), fasting blood glucose and B-HCG serum (pregnancy test). Laboratory tests and electrocardiograms were performed in 3 visits: visit 1 (initial visit), visit 8 (after treatment period) and visit 11 (last visit, after the extension period).

### Statistical method

Based on the bilateral test for proportions, with a significance level of 5%, it was determined that 54 participants per group would provide 80% power. The discontinuation rate was estimated at 20% and, therefore, randomization of 136 participants (68 per group) was planned.

Eligible participants were assigned to receive levetiracetam or placebo using a computer-generated randomization list prepared by an independent biostatistician. The randomization schedule was based on randomly permuted blocks of size four. Participants were stratified according to age (≥ 4 and < 16 years; and ≥ 16 and < 66 years).

The main analysis was performed using the Intention to Treat (ITT) population, which was defined as all the randomized participants who received at least one dose of the products (levetiracetam or placebo). To assess the robustness of the results, efficacy analyses were performed on the population, using a protocol (PP) that was defined as including all the participants in the ITT population who did not violate the inclusion or exclusion criteria, and who did not discontinue treatment before week 16 unless due to adverse events or a clinical need to change the basic therapy, with adherence greater than 80% and no major protocol violations. The safety population was defined using the same criteria as the ITT population.

A logistic regression model was used to analyze the primary and secondary variables involved in the therapeutic response. The following were included in the model: treatment, age range according to stratification, treatment versus age interaction and baseline and center values ​​as covariates. Estimates and 95% confidence intervals (95% CI) for the risk ratio between treatments were obtained. The absolute variation in the average number of focal seizures per week and the average number of days with focal seizures of each subtype (IA, IB and IC) were evaluated as secondary variables of effectiveness, by means of the Wilcoxon rank sum test.

The last observation carried forward (LOCF) estimation was used only for one participant in the levetiracetam group who used the drug for which this individual had been randomized but did not make any subsequent journal entries. The analyses were performed using the SAS V 9.2 system (Statistical Analysis System, SAS Institute) and the bilateral significance level was taken to be equal to 5%.

## RESULTS

### Demographics and baseline characteristics

**Figure 2.**
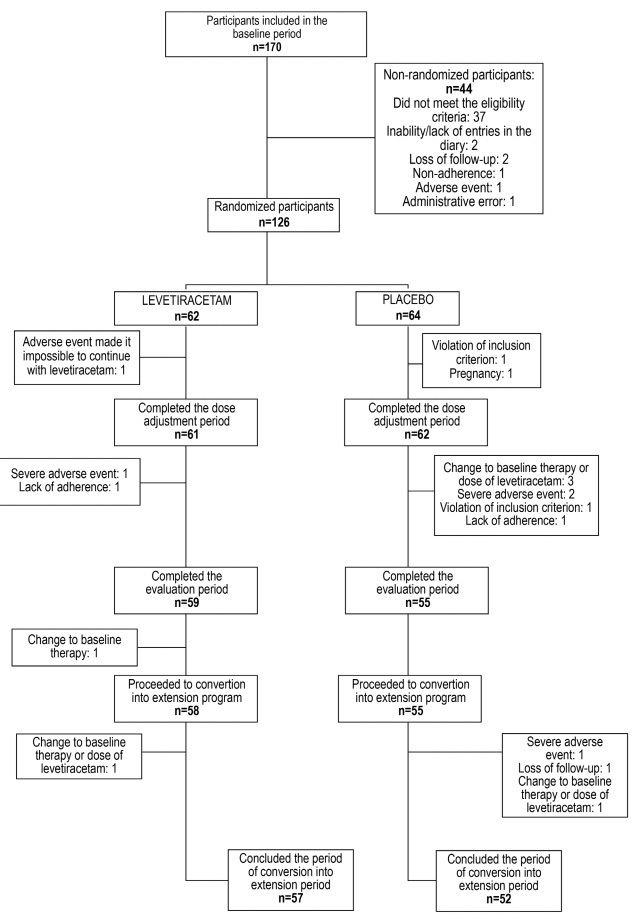
Distribution of the participants in the study

At the end of the study, 114 participants had completed the 16 weeks of treatment: 59 (95.2%) with levetiracetam and 55 (85.9%) with placebo (Figure 2).

The baseline characteristics of the participants are described in Table 1.

**Table 1.** Demographic characteristics and antiepileptic drugs used for the Intention to Treat population.

		Levetiracetam n = 62 (%)	Placebo n = 63 (%)	Total n = 125 (%)
Sex	Male	38 (61.3)	32 (50.8)	70 (56.0)
	Female	24 (38.7)	31 (49.2)	55 (44.0)
Age (years)	Mean (SD)	23.63 (15.35)	25.49 (15.91)	24.57 (15.60)
Age group	4 to 15 years	28 (45.16)	29 (46.03)	54 (45.6)
	Above 15 years	34 (54.84)	34 (53.97)	68 (54.4)
Race	White	43 (69.4)	44 (69.8)	87 (69.6)
	Nonwhite	19 (30.7)	19 (30.1)	38 (30.4)
Time (month) under therapeutic regimen	Mean (SD)	6.48 (7.65)	7.13 (9.35)	6.81 (8.52)
No. of antiepileptic drugs in the therapeutic regimen	1	6 (9.7)	6 (9.5)	12 (9.6)
	2	30 (48.4)	26 (41.3)	56 (44.8)
	3	26 (41.9)	31 (49.2)	57 (45.6)
Antiepileptic drugs used	CLB	29 (46.8)	34 (54.0)	63 (50.4)
	CBZ	35 (56.5)	22 (34.9)	57 (45.6)
	OCBZ	12 (19.4)	19 (30.2)	31 (24.8)
	LTG	12 (19.4)	15 (23.8)	27 (21.6)
	VPA	14 (22.6)	13 (20.6)	27 (21.6)
	TPM	13 (21.0)	13 (20.6)	26 (20.8)
	PB	10 (16.1)	12 (19.0)	22 (17.6)
	PHT	6 (9.7)	6 (9.5)	12 (9.6)
	CNZ	4 (6.5)	6 (9.5)	10 (8.0)
	DVPA	3 (4.8)	7 (11.1)	10 (8.0)
	NZP	5 (8.1)	0 (0.0)	5 (4.0)
	VGB	1 (1.6)	1 (1.6)	2 (1.6)
	ESM	0 (0.0)	1 (1.6)	1 (0.8)
	PRM	0 (0.0)	1 (1.6)	1 (0.8)
	Others	0 (0.0%)	1 (1.6%)	1 (0.8)

n (%): number and percentage of participants in relation to the total treatment group; SD: standard deviation; CLB: clobazam; CBZ: carbamazepine; OCBZ: oxcarbazepine; LTG: lamotrigine; VPA: sodium valproate; TPM: topiramate; PB: phenobarbital; PHT: phenytoin; CNZ: clonazepam; DVPA: divalproex sodium; NZP: nitrazepam; VGB: vigabatrin; ESM: ethosuximide; PRM: primidone.

Regarding the characteristics of seizures, it was observed that focal seizures with impaired awareness were more frequent, affecting 91.9% of the participants in the levetiracetam group and 88.9% of the participants in the placebo group (Table 2).

**Table 2.** Characteristics of the participants in relation to focal epileptic seizures during the baseline period, for the Intention to Treat population.

	Focal seizures (IA + IB + IC)		Focal aware seizures (lA)		Focal seizure with impaired awareness (lB)		Focal to bilateral tonic-clonic seizure (lC)	
	Levetiracetam (n = 62)	Placebo (n = 63)	Levetiracetam (n = 62)	Placebo (n = 63)	Levetiracetam (n = 62)	Placebo (n = 63)	Levetiracetam (n = 62)	Placebo (n = 63)
Frequency of participants with seizures, n (%)	62 (100.0%)	63 (100.0%)	26 (41.9%)	23 (36.5%)	57 (91.9%)	56 (88.9%)	27 (43.5%)	26 (41.3%)
Frequency of seizures/week*								
Median (Q1-Q3)	3 (2-8)	4 (2-10)	0 (0-1)	0 (0-1)	2 (1-4)	2 (1-5)	0 (0-1)	0 (0-1)
Mean (SD)	6.18 (7.22)	8.22 (10.22)	1.03 (2.45)	2.34 (6.25)	4.04 (5.14)	4.75 (7.13)	1.10 (3.42)	1.13 (2.82)
Frequency of days with seizures/week**								
Median (Q1-Q3)	2 (1-4)	2 (1-5)	0 (0-0)	0 (0-1)	2 (1-3)	2 (1-3)	0 (0-0)	0 (0-1)
Mean (SD)	2.76 (1.87)	2.99 (2.06)	0.63 (1.33)	0.92 (1.92)	2.13 (1.90)	2.12 (1.94)	0.58 (1.36)	0.55 (1.14)

IA: focal aware seizures; IB: focal seizures with impaired awareness; IC: focal to bilateral tonic-clonic seizures; n (%): number and percentage of study participants who presented at least one episode of epileptic seizure during the baseline period (week 1 to week 8); this percentage was established in relation to the number of participants in the treatment group; SD: standard deviation; Min-Max: minimum and maximum values observed; Q1 and Q3: 25^th^ and 75^th^ percentiles; *calculated as the ratio between the total number of seizures and the number of days evaluated during the baseline period, multiplied by 7; **calculated as the ratio between the total number of days with epileptic seizures and the number of days evaluated during the baseline period, multiplied by 7

### Efficacy evaluation

Regarding the primary outcome, 38.7% of the levetiracetam group and 14.3% of the placebo group showed reductions in the mean number of focal seizures/week ≥ 50% (Table 3).

**Table 3.** Proportion of responders with reduction ≥ 50% regarding the average number of focal seizures/week without treatment period, for the Intention to Treat population.

Age range		Levetiracetam	Placebo	Variation
Total	n (%)	24/62 (38.71%)	9/63 (14.29%)	24.42%
	95% CI (%)	26.60-51.90%	6.75-25.40%	6.57-40.30%

n (%): number and proportion of responders with therapeutic response in each treatment group; 95% CI: 95% confidence intervals for proportion of responders with therapeutic response.

**Figure 3.**
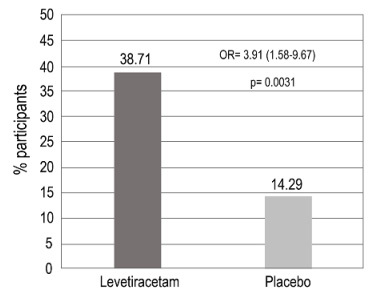
Proportion of participants with a reduction ≥ 50% in the mean number of focal epileptic seizures/week during the treatment period, for the Intention to Treat population.

The estimation of the risk ratio indicated that the chance of ≥ 50% reduction in the mean number of focal seizures/week at the end of treatment for participants in the levetiracetam group was 3.91 times higher than in the placebo group (Figure 3).

**Figure 4.**
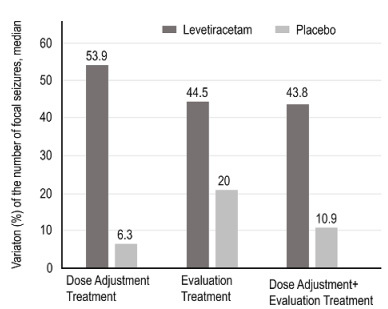
Median percentage variation of the mean number of epileptic seizures/week for each study period in relation to the baseline period, for the Intention to Treat population.

The median percentage change in the average number of focal seizures per week for the different study periods in relation to the baseline period is shown in Figure 4.

In the analysis of secondary variables, the absolute variation from baseline for each subtype is summarized according to treatment group and study period in Table 4.

**Table 4.** Variation of the mean number of seizures or days with focal seizures/week for each subtype, for the Intention to Treat population.

	Levetiracetam (n = 62)		Placebo (n = 63)	
	Mean (SD)	Median (Q1-Q3)	Mean (SD)	Median (Q1-Q3)
Mean number of seizures/week				
Focal seizures (IA + IB + IC + III)	2.33 (4.20)	1.2 (0.3-3.1)	0.61 (4.02)	0.4 (-0.4-2.0)
Focal aware seizures (IA)	0.52 (2.13)	0.0 (0.0-0.3)	0.39 (1.68)	0.0 (0.0-0.4)
Focal seizure with impaired awareness (IB)	1.29 (3.55)	0.7 (0.1-1.7)	0.46 (3.05)	0.2 (-0.4-1.0)
Focal to bilateral tonic-clonic (IC)	0.52 (2.03)	0.0 (0.0-0.4)	-0.24 (1.95)	0.0 (0.0-0.1)
Mean number of days/week				
Focal seizures (IA + IB + IC + III)	0.72 (1.01)	0.7 (0.1-1.3)	0.13 (1.06)	0.2 (-0.1-0.8)
Focal aware seizures (IA)	0.33 (1.05)	0.0 (0.0-0.3)	0.22 (0.62)	0.0 (0.0-0.2)
Focal seizure with impaired awareness (IB)	0.62 (0.94)	0.6 (0.0-1.2)	0.05 (0.97)	0.1 (-0.3-0.5)
Focal to bilateral tonic-clonic (IC)	0.19 (0.67)	0.0 (0.0-0.2)	-0.04 (0.47)	0.0 (0.0-0.1)

Variation: mean number of days or seizures/week during the baseline period - mean number of days or seizures/week during the treatment period; SD: standard deviation; Q1 and Q3: 25^th^ and 75^th^ percentiles.

At the baseline, the medians of the average number of focal seizures with impaired awareness per week were 2.4 and 2.3 for the levetiracetam and placebo groups, respectively; and in the treatment period, these were 1.0 and 2.1, respectively. The medians of the average number of days with focal seizures with impaired awareness per week during the baseline period were 1.6 and 1.5 in the levetiracetam and placebo groups, respectively. On the other hand, the median for focal aware seizures or focal to bilateral tonic-clonic seizure was zero.

A statistically significant difference was observed between the treatment groups regarding the number of focal seizures with impaired awareness (p = 0.0031), but there was no statistically significant difference between treatments regarding the subtypes of focal aware seizures (p = 0.4046) and focal to bilateral tonic-clonic seizures (p = 0.1397).

**Figure 5.**
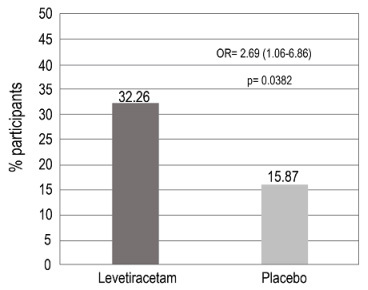
Proportion of the participants with reduction ≥ 50% in the mean number of days/week of focal epileptic seizures during the treatment period, for the Intention to Treat population.

The proportion of the responder participants with ≥ 50% reduction in the average number of days/week with focal seizures during the treatment period was 20 (32.3%) participants in the levetiracetam group and 10 (15.9%) participants in the placebo group, The difference between the groups was 16.4% (95%CI -1.52- 32.8; p = 0.0382) (Figure 5).

The proportion of the participants free from focal seizures during the evaluation period was estimated at 7.0% (95% CI 2.0-17.0) in the levetiracetam group and 5.7% (95%CI 1.2-15.7) in the placebo group, considering the ITT population. There was no difference between the treatment groups in this regard.

Comparison between groups in relation to QOLIE-31 showed that there was no statistically significant difference between the treatments. QOLIE AD-48 showed a statistically significant difference regarding the impact of epilepsy (p = 0.0255) and total score (p = 0.0362), thus indicating a slight improvement for the placebo group.

There was no statistically significant effect from treatment regarding the response to the NDDI-E Depression Inventory at week 24 (p = 0.1716), in an evaluation using the logistic regression model and considering the classification of week 8 as the covariate.

Treatment adherence was above 90% in both groups.

### Safety assessment

Adverse events with an incidence greater than or equal to 5% are described in Table 5.

**Table 5.** Adverse effects according to systems and organs (Medical Dictionary for Regulatory Activities).

Organs and systems (MedDRA)	Treatment (Week 9-Week 24)	
	Levetiracetam n = 62 (%)	Placebo n = 63 (%)
Nervous system complaints	19 (30.6)	20 (31.7)
Gastrointestinal complaints	10 (16.1)	11 (17.5)
Infections and infestations	12 (19.4)	12 (19.0)
Psychiatric disturbances	10 (16.1)	8 (12.7)
Respiratory, thoracic and mediastinal diseases	4 (6.5)	8 (12.7)
Complications of interventions relating to injuries and intoxications	6 (9.7)	6 (9.5)
General disturbances on the administration site	4 (6.5)	8 (12.7)
Cutaneous and subcutaneous tissue conditions	5 (8.1)	2 (3.2)
Musculoskeletal and conjunctive tissue conditions	2 (3.2)	4 (6.3)
Metabolism and nutrition-related diseases	2 (3.2)	5 (7.9)
Ear and labyrinth diseases	2 (3.2)	3 (4.8)
Eye conditions	3 (4.8)	2 (3.2)
Vascular disorders	1 (1.6)	4 (6.3)

MedDRA: Medical Dictionary for Regulatory Activities.

In all periods of the study, adverse events of mild intensity predominated. These were unrelated to the study drug, with no need for medication adjustments, and they had all resolved by the end of the study. During the treatment period, medication to treat adverse events was more frequently needed in the levetiracetam group (69.1%) than in the placebo group (52.4%).

No difference was found between the groups regarding vital signs such as blood pressure, heart rate and respiratory rate, and no abnormal and clinically significant vital signs were recorded.

## DISCUSSION

Levetiracetam is a broad-spectrum ASM that is recommended as a first-line add-on agent for focal seizures, with a favorable profile of efficacy and safety for both children and adults. It is one of the most-prescribed new-generation ASMs^12^.

Previous studies have proven the efficacy and safety of LEV in relation to focal seizures in both adults and children.

In a systematic review evaluating the use of levetiracetam among children with focal onset seizures, levetiracetam had a mean response rate of 56% occurrence of adverse events, which was comparable to placebo, with a low discontinuation rate^13^.

In a meta-analysis that included a total of 3,205 participants (both children and adults), a reduction of 50% from baseline was reported, and the results suggested that patients treated with leveti racetam had a substantially higher responder rate than did those who received placebo (RR = 2.17; 95% CI 1.93-2.43; p = 0.05). Use of 2,000 mg/day showed the best efficacy and safety ratio. There was a 75% reduction in seizures through using LEV, with similar results for doses of 2,000 and 3,000 mg. The safety of LEV was comparable to that of placebo^7^.

Three pivotal studies have demonstrated that levetiracetam at doses of 1,000-3,000 mg/day is effective as add-on therapy among adults with refractory focal seizures^14^^,^^15^^,^^16^. The European Levetiracetam Study Group showed that there was a significant reduction in seizure frequency, of ≥ 50%, in 22.8% and 31.6% of patients in the 1,000 and 2,000 mg groups, respectively, compared to placebo (10.4%), with no significant difference in the incidence of adverse events between the groups^14^.

In our study, we explored the efficacy, tolerability and safety profile of LEV in the Brazilian population. We showed that its use gave rise to large reductions of at least 50% in the average number of focal seizures per week in 38.7% of the LEV group and 14.3% of the placebo group, with statistically significance (p = 0.0031), while no significant difference in adverse events was found between the groups. Treatment adherence was above 90% in both groups.

Although several studies have shown that levetiracetam as an adjuvant therapy positively influences health-related quality of life^13^, the present study did not show any statistically significant difference between the groups regarding QOLIE-31^9^ variation. Most likely, this assessment was hampered by the small number of study participants. QOLIE AD-48^10^ showed differences regarding the impact of epilepsy (p = 0.0255) and the total score (p = 0.0362), which indicated a slight improvement for the placebo group. This result may have been influenced by caregivers’ perceptions regarding participants under the age of 18 years.

In summary, the findings from this study demonstrate that levetiracetam at doses of 1,000-3,000 mg/day or 60 mg/kg/day (children) is an effective and safe ASM for patients with refractory focal epilepsy, both among Brazilian children over 4 years old and among adults.
